# Pretreatment malnutrition and quality of life - association with prolonged length of hospital stay among patients with gynecological cancer: a cohort study

**DOI:** 10.1186/1471-2407-10-232

**Published:** 2010-05-25

**Authors:** Brenda Laky, Monika Janda, Srinivas Kondalsamy-Chennakesavan, Geoffrey Cleghorn, Andreas Obermair

**Affiliations:** 1 Queensland Centre for Gynaecological Cancer, Level 6 Ned Hanlon Building, The Royal Brisbane and Women's Hospital, Herston Queensland 4029, Australia; 2 School of Public Health, Queensland University of Technology, Brisbane, Australia; 3 Children's Nutrition Research Centre, Royal Children's Hospital, Brisbane, Australia; 4 School of Medicine, University of Queensland, Brisbane, Australia

## Abstract

**Background:**

Length of hospital stay (LOS) is a surrogate marker for patients' well-being during hospital treatment and is associated with health care costs. Identifying pretreatment factors associated with LOS in surgical patients may enable early intervention in order to reduce postoperative LOS.

**Methods:**

This cohort study enrolled 157 patients with suspected or proven gynecological cancer at a tertiary cancer centre (2004-2006). Before commencing treatment, the scored Patient Generated - Subjective Global Assessment (PG-SGA) measuring nutritional status and the Functional Assessment of Cancer Therapy-General (FACT-G) scale measuring quality of life (QOL) were completed. Clinical and demographic patient characteristics were prospectively obtained. Patients were grouped into those with prolonged LOS if their hospital stay was greater than the median LOS and those with average or below average LOS.

**Results:**

Patients' mean age was 58 years (SD 14 years). Preoperatively, 81 (52%) patients presented with suspected benign disease/pelvic mass, 23 (15%) with suspected advanced ovarian cancer, 36 (23%) patients with suspected endometrial and 17 (11%) with cervical cancer, respectively. In univariate models prolonged LOS was associated with low serum albumin or hemoglobin, malnutrition (PG-SGA score and PG-SGA group B or C), low pretreatment FACT-G score, and suspected diagnosis of cancer. In multivariable models, PG-SGA group B or C, FACT-G score and suspected diagnosis of advanced ovarian cancer independently predicted LOS.

**Conclusions:**

Malnutrition, low quality of life scores and being diagnosed with advanced ovarian cancer are the major determinants of prolonged LOS amongst gynecological cancer patients. Interventions addressing malnutrition and poor QOL may decrease LOS in gynecological cancer patients.

## Background

Despite significant advances in cancer therapy, in-patient hospital care remains a major expense in the treatment of patients with gynecological cancer. Reducing length of hospital stay (LOS) has the potential to decrease health care cost, risk of infections and other hospital acquired diseases, and to improve patients' quality of life (QOL). Previous studies have observed differential LOS by cancer type and stage, however, these characteristics commonly are not available before surgery, and thus are not amenable to pre-treatment interventions. Identifying modifiable risk factors at admission predicting (LOS) could lead to appropriately targeted interventions to improve the delivery of care for women with gynecological cancer. Previous research has shown that preoperative serum albumin (a marker of chronic malnutrition), hemoglobin and lymphocyte status are associated with LOS and mortality in the gynecological oncology setting [1-4]. Preoperative medical conditions or co-morbidities could also be associated with LOS; however findings regarding these predictors are inconsistent [4,5].

Acute malnutrition may be a further potentially amenable risk factor for LOS. Malnutrition has been found to be associated with increased risk of morbidity and mortality, complication rates such as wound infections, costs of hospitalisation, and decreased QOL in various cancer populations [2,4,6-16]. The scored Patient Generated - Subjective Global Assessment (PG-SGA) is a validated nutritional assessment tool specifically designed for cancer patients. Using the PG-SGA, we have observed high levels of nutritional deficiencies among patients with gynecological cancer, especially those diagnosed with ovarian cancer [17,18], but to date no study has assessed whether the scored PG-SGA can predict prolonged LOS among gynecological cancer patients.

Gynecological cancer and/or malnutrition can have a profound impact on patients' physical function and psychosocial well-being - both important components of QOL. One common definition of QOL is that it is a subjective, multidimensional construct representing functional status, mental and social well-being as well as general health [19]. Health-related QOL before treatment commences has been suggested as an important prognostic factor in ovarian cancer patients [20,21]. To our knowledge, no study to date evaluated the association between preoperative QOL, malnutrition and LOS among a wider range of gynecological cancer patients.

Thus, the purpose of this study was to evaluate factors available prior to initial treatment to predict LOS in patients with suspected or proven gynecological cancer.

## Methods

The study was conducted at the Queensland Centre for Gynecological Cancer at the Royal Brisbane and Women's Hospital, Brisbane, Australia. Patients with presumed or proven primary gynecological cancer were screened for eligibility at their preoperative visit between March 2004 and December 2006. Patients were excluded from the study if they presented with recurrent cancer, had received treatment for other cancers within the past five years, had psychological or cognitive impairments (e.g. schizophrenia, dementia) or were non-English speaking. A total of 194 patients gave informed written consent. Thirty-two study participants declined to complete the Functional Assessment of Cancer Therapy-General (FACT-G) questionnaire and five participants had to be excluded from analyses due to incomplete data. Overall, 157 women completed both the scored PG-SGA and the FACT-G questionnaire before commencing treatment. Patients' clinical and demographic characteristics including age at study entry, body mass index, serum albumin, hemoglobin, lymphocytes, co-morbidities, adverse events, surgical approach, LOS, and histopathological diagnosis and staging according to the International Federation of Gynecology and Obstetrics (FIGO) were recorded prospectively. Six women did not undergo surgery, because a surgical approach was contraindicated or chemotherapy and/or radiotherapy was initiated as the primary treatment. Pre-treatment serum albumin levels were recorded in 146 patients; hemoglobin and lymphocyte count in 153 patients and actual body weight and height for all but one patient. All pre-treatment predictive factors were collected either at the outpatient or preadmission clinic, typically one to five weeks before primary treatment was initiated. The median waiting time for surgery was two weeks for women with suspected ovarian and cervical cancers and five weeks for women with suspected benign diseases or endometrial cancer, respectively. Women were categorised according to their clinical diagnosis before surgery: pelvic mass/benign disease, suspected advanced ovarian cancer, cervical cancer or endometrial cancer. This study has been approved by The Royal Brisbane and Women's Hospital Human Research Ethics Committee (Protocol Number 2004/007) and the University of Queensland Medical Research Ethics Committee (Brisbane, Australia; Project Number 2006000533).

### Nutritional assessment

The scored PG-SGA is a validated nutritional assessment tool for cancer patients [9,16] that records and summarizes weight changes, alterations in food intake, gastrointestinal symptoms (such as nausea, vomiting and diarrhoea that have persisted for two weeks), and changes in functional capacity and physical signs of malnutrition. These signs were assessed by a trained clinical dietician using skinfold measurements (loss of subcutaneous fat, muscle wasting, edema). The presence of ascites was established radiologically and abstracted from the clinical notes for this study. Based on the global rating, women were classified as well nourished (PG-SGA A); moderately malnourished or suspected of being malnourished (PG-SGA B); or severely malnourished (PG-SGA C). The PG-SGA also incorporates a numerical score (PG-SGA score). Typical scores achieved by gynaecological cancer patients range from 0-28 [22], with higher scores reflecting greater risk of malnutrition. Scores of nine or more indicate a critical need for nutritional intervention options and/or improved symptom management [23].

### QOL assessment

QOL was measured by the FACT-G questionnaire, which is a widely utilized and validated instrument [24]. Version 4 of the FACT-G is a 27-item self-report measure and allows patients to rate their current physical (7 items, range: 0-28), social and family (7 items, range: 0-28), emotional (6 items, range: 0-24), and functional well-being (7 items, range: 0-28). The FACT-G is scored using a 5-point scale (0 = not at all, 1 = a little bit, 2 = somewhat, 3 = quite a bit, 4 = very much). The raw scores were are linearly transformed as per questionnaire manual with higher scores indicating better QOL [25].

### Statistical analysis

Descriptive statistics were used to summarize patient characteristics and to group patients by the main outcome variable (average versus prolonged LOS). Prolonged LOS was defined as LOS of greater than five days for patients who underwent open abdominal surgery and greater than two days for patients who underwent vaginal of laparoscopic surgery. Univariate logistic regression analyses were conducted to calculate odds ratios adjusted for age and surgical approach for patients who had prolonged compared to average LOS.

Variables significantly associated with LOS were then entered into a multivariable model (Table 1).

**Table 1:** Pre-surgical factors associated with prolonged length of hospital stay (LOS).

		% (n) with	Univariate model^1^			Multivariable model^2^		
	N	prolonged LOS	OR	95% CI	*P*	OR	95% CI	*P*
Total	157		48.1 (75)					
*Body mass index*								
Underweight (<18.5)	3	66.7 (2)	2.96	0.12 - 70.3	0.50			
Normal (18.5-24.99)	39	46.2 (18)	**Reference group**					
Overweight (25.0-29.99)	47	44.7 (21)	0.91	0.36 - 2.30	0.84			
Obese (≥ 30)	67	50.7 (34)	1.31	0.55 - 3.16	0.54			
*Pre-existing co-morbidity*								
No or 1 co-morbidity	107	45.8 (49)	**Reference group**					
2 or more co-morbidities	50	54.0 (27)	0.12	0.56 - 2.75	0.59			
*Blood*								
Albumin								
Average (>35 g/L)	124	41.9 (52)	**Reference group**					
Below Average (≤ 35 g/L)	22	86.4 (19)	8.81	1.82 - 42.6	0.007	0.86	0.12 - 6.5	0.88
Hemoglobin								
Average (>120 g/L)	117	42.7 (50)	**Reference group**					
Below Average (≤ 120 g/L)	36	69.4 (25)	3.21	1.31-7.68	0.01	2.11	0.65-6.86	0.21
Lymphocytes								
Average (1.5-4.0 × 10^9^/L)	109	44.0 (48)	**Reference group**					
Below Average (≤ 1.5 × 10^9^/L)	44	64.4 (27)	1.46	0.66 - 3.22	0.35			
*FACT-G global score*	157	[75.3 ± 16.9]^4^	0.95	0.93 - 0.98	<0.001	0.96	0.93 - 0.99	0.007
*Nutritional assessment*								
PG-SGA score	157	[9.5 ± 6.6]^4^	1.13	1.05 - 1.22	0.001	0.94	0.84 - 1.06	0.33
PG-SGA A	118	39.3 (44)	**Reference group**					
PG-SGA B and C	39	82.1 (32)	6.89	2.48 - 19.2	<0.001	5.28	0.98 - 28.5	0.05
*Histology and Stage*								
Benign/LMP/OvCa stage I or II^4^	81	42.0 (34)	**Reference group**					
OvCa stage III or IV	23	95.7 (22)	30.4	3.9-236.7	0.001	14.7	1.6-134.7	0.02
Endometrial cancer	36	50.0 (18)	1.38	0.63-3.04	0.42	1.71	0.67-4.34	0.07
Cervical cancer	22	11.8 (2)	0.18	0.04-0.86	0.03	0.10	0.01-0.71	0.84

^1^adjusted for age and surgical approach; ^2 ^Multivariable model including albumin, FACT-G score, PG-SGA score and PG-SGA global rating; adjusted for age and surgical approach; ^3 ^Continuous variables: mean ± standard deviation for group with average LOS; ^4^Continuous variables: mean ± standard deviation for group with prolonged LOS;

*Abbreviations: *LOS, Length of hospital stay; OR, Odds Ratio; CI, Confidence interval; PG-SGA, Patient-Generated Subjective Global Assessment; FACT-G, Functional Assessment of Cancer Therapy-General.

All models were adjusted for age and surgical approach (open-abdominal, vaginal or laparoscopic surgery). Hosmer-Lemeshow goodness-of-fit statistic assessed fit of the models. SPSS software version 16.0 Graduate Student (SPSS Inc., Chicago, IL, USA) was used for the statistical analyses.

## Results

### Patient Characteristics

Patients' mean age was 58 years (SD 14 years). Preoperatively, 81 (52%) patients presented with suspected benign disease/pelvic mass, 23 (15%) were suspected to have advanced ovarian cancer, 36 (23%) patients to have endometrial cancer, and 17 (11%) patients to have cervical cancer. Ninety-four patients were operated using an open-abdominal, 40 patients with a laparoscopic and 17 patients with a vaginal surgical approach. Data on patients' weight, body mass index, albumin and PG-SGA have been reported in previous publications [17,22].

### LOS and potential predictors

The median LOS for all patients in the study was 5 days (range 0-43 days). In total, 75 (48.1%) of the patients had prolonged LOS (Table 1). Patients with prolonged LOS were significantly older (mean age 62.8 years, SD 13.7) than patients with average LOS (mean age 54.5 years, SD 13.6) (p < 0.01).

BMI was not significantly associated with LOS. Although fifty patients had two or more pre-existing medical co-morbidities, neither the number (Table 1), nor the specific type of co-morbidity (data not shown), were significantly different between patients with average and prolonged LOS. Low pretreatment serum albumin or hemoglobin levels, low QOL FACT-G scores and high PG-SGA scores were associated with prolonged LOS. Compared to patients with a suspicious pelvic mass, patients suspected to carry an advanced ovarian cancer were significantly more likely to have prolonged LOS (OR = 30.4; 95% CI = 3.9-236.7) (Table 1).

In the multivariable model, patients with good QOL were at significantly lower risk for prolonger LOS (OR = 0.96, 95% CI 0.93-0.99; p = 0.007). Patients with PG-SGA B or C (OR = 5.28, 95% CI = 0.98-28.5, p = 0.05) and patients with suspected advanced stage ovarian cancer (OR = 14.7, 95% CI = 1.61-134.7. p = 0.02) were significantly more likely to have prolonger LOS compared to other patients (Table 1).

## Discussion

This study assessed factors predictive for prolonged LOS (defined as >5 days or >2 days, for patients who had open abdominal or laparoscopic surgery, respectively) among gynecological cancer patients. Malnutrition and low QOL were found to predict prolonged LOS independent of patients' age at diagnosis, surgical approach (laparoscopy or laparotomy), albumin and hemoglobin and suspected clinical diagnosis. Patients suspected to have stage III or IV ovarian cancer were also independently at greater risk for prolonged LOS compared to other patients.

Previous studies have shown that malnutrition was associated with prolonged LOS in hospitalized patients requiring treatment for various types of cancer [13,16,26-28]. The present manuscript is the first to confirm those findings for patients with gynecological cancer, and extends these findings even further. The present data suggest an association between pre-operative malnutrition and LOS based on both the PG-SGA global rating and the PG-SGA score. To our knowledge there is only one previous study which assessed the PG-SGA score (as opposed to the global rating) and LOS [15]. Thomas and colleagues found a weak correlation between LOS and the PG-SGA score [15], and similar in our study the PG-SGA score was the weaker predictor compared to the global rating in multivariable analysis. However, the PG-SGA score provides dieticians with suggested appropriate intervention strategies and thus can be a clinically useful adjunct to the global rating.

Our findings show that LOS was not equally distributed between tumor types. Even after adjustment for age and surgical approach, 96% of all suspected advanced stage ovarian cancer patients had prolonged hospital stay, compared with only 50% of patients with endometrial cancer and 42% of patients with a pelvic mass. In our previous work we described in detail that malnutrition was almost exclusively present in ovarian cancer patients compared to other gynecologic oncology diagnoses [17].

A previous study by Dean and colleagues found that gynecological cancer patients with two or more pre-existing co-morbidities had significantly longer LOS than those with one or no co-morbidities [5]. In contrast, our findings suggest that in this sample of gynecological cancer patients, neither the number of co-morbidities nor the type of co-morbidity are predictive of prolonged LOS. Further studies are required to validate these findings, carefully separating preoperative co-morbidities from postoperative complications.

In this study, we found low preoperative hemoglobin and lymphocyte counts in a small number of patients and these patients were more likely to experience prolonged LOS, but this difference was no longer statistically significant in adjusted modelling. Previous studies suggested that low hemoglobin levels were related to poor survival in patients with ovarian cancer [29,30], and studies differed in their conclusions regarding the importance of age, histology, nutritional status and immune system on lymphocyte counts [31-33]. With our non-significant results, we suggest that neither of these markers are suitable factors to predict prolonged LOS.

A third biochemical marker assessed in the present study was serum albumin, generally believed to indicate the presence of chronic malnutrition. Previous studies have demonstrated an association between low serum albumin and increased postoperative complications [6,34,35], as well as reductions in survival in ovarian cancer patients [36]. The results of our study confirmed previous findings by Massad and colleagues in which they found an inverse correlation between serum albumin and LOS among patients with gynecological cancer [4]. However, our current results demonstrated that when albumin and PG-SGA global rating [22] were assessed concurrently, the PG-SGA was a stronger predictor of LOS, and therefore more sensitive to detect malnutrition in gynecological cancer patients.

Research conducted on QOL and nutritional status in cancer patients indicates that nutritional support may lead to better QOL [11,37,38]. To our knowledge few investigations have examined the association between patients' pre-treatment QOL and prolonged LOS, while concurrently assessing malnutrition. One study by Rogers and colleagues found a weak correlation between better QOL and shorter LOS in oral and oropharyngeal cancer patients [39]. In the present study we found that poor QOL prior to initial treatment was associated with prolonged LOS, and this association remained significant when adjusted for other variables. Our results suggest that subsequent studies should assess the effect of pre-surgical nutritional interventions, or interventions aimed at improving QOL on LOS. A limitation of our study was that data on nutritional status and QOL were only assessed before commencement of initial treatment and not at any time point afterwards. This additional information would have been of interest to identify whether changes in patients' PG-SGA score and global rating and FACT-G score are more influential for LOS than pre-treatment score alone. In addition, given the preponderance of malnutrition among ovarian cancer patients, subsequent studies should focus on this subgroup of gynecological cancer patients. However, our study also has some major strengths, including the availability of pre-treatment assessments for all patients, and the high participation rate among patients with gynecological cancer.

## Conclusions

In conclusion, prolonged LOS was found to be associated with low presurgical QOL and malnutrition. Strategies to reduce LOS and improve patients' well-being during hospital stay will need to address these potentially modifiable factors, particularly among women with suspected advanced ovarian cancer.

## Competing interests

The authors declare that they have no competing interests.

## Authors' contributions

The authors' responsibilities were as follows - AO: initiated the study; BL, SK: collected data; AO, BL, and MJ: data analysis; AO and GC: project supervision; AO, BL, SK and MJ: writing the manuscript; and all authors: revision of the manuscript.

## Pre-publication history

The pre-publication history for this paper can be accessed here:

http://www.biomedcentral.com/1471-2407/10/232/prepub
